# Label-Free Single-Particle Imaging of the Influenza Virus by Objective-Type Total Internal Reflection Dark-Field Microscopy

**DOI:** 10.1371/journal.pone.0049208

**Published:** 2012-11-15

**Authors:** Sawako Enoki, Ryota Iino, Nobuhiro Morone, Kunihiro Kaihatsu, Shouichi Sakakihara, Nobuo Kato, Hiroyuki Noji

**Affiliations:** 1 Department of Applied Chemistry, School of Engineering, The University of Tokyo, Bunkyo-ku, Tokyo, Japan; 2 CREST, Japan Science and Technology Agency, Chiyoda-ku, Tokyo, Japan; 3 Institute for Integrated Cell-Material Sciences, Kyoto University, Sakyo-ku, Kyoto, Japan; 4 Institute of Scientific and Industrial Research, Osaka University, Ibaraki, Osaka, Japan; University of Edinburgh, United Kingdom

## Abstract

Here we report label-free optical imaging of single particles of the influenza virus attached on a glass surface with a simple objective-type total internal reflection dark-field microscopy (TIRDFM). The capability of TIRDFM for the imaging of single viral particles was confirmed from fine correlation of the TIRDFM images with fluorescent immunostaining image and scanning electron microscopy image. The density of scattering spots in the TIRDFM images showed a good linearity against the virus concentration, giving the limit of detection as 1.2×10^4^ plaque-forming units per milliliter. Our label-free optical imaging method does require neither elaborated sample preparation nor complex optical systems, offering a good platform for rapid and sensitive counting of viral particles.

## Introduction

The rapid detection of viruses, especially the influenza virus, in the early stage of infection is important for preventing pandemics. Conventional methods of virus detection include the plaque-formation assay of infective viruses on cultured mammalian cells, the enzyme-linked immunosorbent assay (ELISA) of viral proteins, and the amplification of viral genomes by the polymerase chain reaction. Another approach that is useful for virus detection is the optical imaging of single viral particles under an optical microscope. Single viral particles can be imaged with fluorescent-labeled antibodies by using a fluorescence microscope. Although fluorescence imaging enables quantitative estimation of the virus concentration in a sample by counting single particles, it is time consuming and is hampered by the photobleaching of fluorescent dyes.

The optical imaging of a single influenza viral particle without labeling may provide great benefits for rapid diagnosis. However, label-free imaging of single viral particles with conventional optical microscopy is difficult because of their small size (∼100 nm) and small difference in refractive index between viral particles and surrounding medium. Thus far, several methods of label-free optical imaging for single viral particles have been reported, including interferometric measurement [1]–[3] and surface plasmon resonance imaging [4]. Although these methods are sophisticated and clear images of the viral particles have been obtained, they have not been used widely for virus detection because of the complexity of the optical system.

Here, we report a method for label-free imaging of single influenza virus particles by using a simple dark-field microscopy. In the present work, we employed objective-type total internal reflection dark-field microscopy (TIRDFM), which has been previously developed and used for high-speed single-particle imaging of colloidal gold attached to single motor proteins [5], [6]. TIRDFM does not require a complex optical system; it can be achieved by just replacing the dichroic mirror used in conventional objective-type total internal reflection fluorescence microscopy (TIRFM) with a perforated mirror. The intensity of light scattered by a single viral particle attached to the glass surface and illuminated with an evanescent field was sufficiently high for real-time imaging with the TIRDFM system equipped with a complementary metal-oxide semiconductor (CMOS) camera. The capability of TIRDFM to obtain scattering images of single viral particles (and not particle aggregates) was confirmed by the correlation of the images with those obtained by TIRFM and scanning electron microscopy (SEM). The density of the scattering spots in the image obtained was proportional to the virus concentration, which enabled quantitative estimation with a limit of detection (LOD) of 1.2×10^4^ plaque-forming units per milliliter (pfu/mL). Our label-free method will offer a good platform for rapid and sensitive counting of viral particles.

## Materials and Methods

### Ethics Statement

This study was approved by Review Board of the institute of scientific and industrial research at Osaka University and Review Board of the graduate school of engineering at University of Tokyo.

### Sample Preparation

Influenza A/Puerto Rico/8/34 (H1N1) was inoculated (50 pfu/egg) into the allantoic fluid of 11-day-old chicken embryonated eggs for 3 days at 37°C. The allantoic fluid solution was centrifuged at 2,500 *g* for 20 min at 4°C. The supernatant was passed through a membrane filter with a pore size of 0.45 µm. The solution was then subjected to 10–60% sucrose density gradient centrifugation for 2 h at 120,000 *g* at 4°C in a swing-bucket rotor. After the centrifugation was completed, 2-mL aliquots of the gradient starting from the top of the tube were collected manually. Each fraction was analyzed using sodium dodecyl sulfate (SDS) polyacrylamide gels. The solutions from selected fractions were ultracentrifuged for 2 h at 120,000 *g* at 4°C in a fixed-angle rotor, and the pellet was suspended in phosphate-buffered saline (PBS). The concentration of infective influenza A/Puerto Rico/8/34 (H1N1) virus was determined by a plaque-formation assay [7]. Briefly, Madin-Darby canine kidney (MDCK) cell monolayers in a 6-well plate were inoculated with 1.2 mL of influenza A/Puerto Rico/8/34 (H1N1) virus appropriately diluted in PBS supplemented with 0.20% bovine serum albumin (BSA). After 60 min at 37°C, 3 mL of agar overlay medium (Dulbecco’s modified Eagle medium [DMEM] containing 0.8% Oxoid agar No. 1, 0.0006% trypsin, and 0.2% BSA) was added to the cultures. After incubation for 2 days at 37°C in a 5% CO_2_ incubator, the cell sheets were stained with methylene blue solution and the plaques formed in each well were counted.

A goat anti-influenza A (H1N1) polyclonal antibody (Fitzgerald Industries International, Acton, MA, USA) was reacted with Cy3 conjugated with *N*-hydroxysuccinimide ester (Cy3-NHS). After incubation for 90 min, non-reacted Cy3-NHS was removed using a desalting column (NAP-5; GE Healthcare, Little Chalfont, UK).

### Setup for TIRDFM and TIRFM

**Figure 1:**
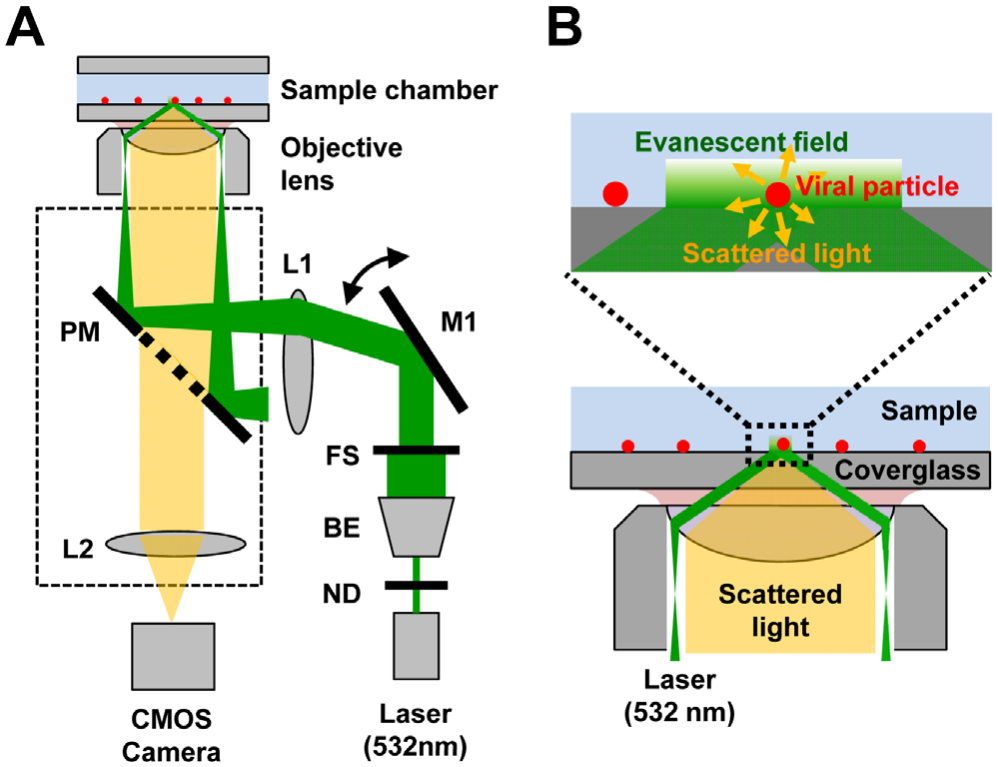
Experimental setup for label-free imaging of viral particles. (A) Schematic representation of the optical system of objective-type TIRDFM. ND, neutral density filter; BE, beam expander; FS, field stop; M1, mirror; L1, lens; PM, perforated mirror; L2, second objective lens inside microscope. (B) Expanded drawings depicting the sample chamber and objective lens.

Figure 1 shows a schematic drawing of the optical system for objective-type TIRDFM. Dark-field imaging with TIRDFM was performed mostly as previously described [5]. An inverted microscope (IX71; Olympus, Tokyo, Japan) was used, and illumination by an evanescent filed was provided using a 532-nm laser (DPGL-2100F; Photop Suwtech, Shanghai, China). The collimated incident laser beam was reflected using a perforated mirror (PM), and a lens (L1) was used to focus the beam onto the back focal plane of the objective lens (APON60X0TIRF, numerical aperture = 1.45, Olympus, Tokyo, Japan). By introducing the laser beam at the edge of the objective lens, total internal reflection occurred at the interface between glass coverslip and medium, and the evanescent field was formed on the glass surface. The light scattered by the viral particle was collected by the same objective lens, passed through the center of a perforated mirror, and projected onto a CMOS camera (FASTCAM-1024 PCI, Photron, Tokyo, Japan) by a lens (L2) and recorded at 500 frames/s. The perforated mirror had an elliptical antireflective surface (minor axis, 2.47 mm; major axis, 3.5 mm; circular window viewed from the optical axis) in its central region for transmitting scattered light through this area. The laser power was set at 6.3 µW/µm^2^ in front of the objective lens. For fluorescence imaging of the same field of view, the perforated mirror was replaced with a dichroic mirror (DM550, Olympus) and an emission filter (HQ590/75 M, Olympus), and objective-type TIRFM was performed. The laser power was set at 6.3 µW/µm^2^ in front of the objective lens. Fluorescence images were obtained with same CMOS camera at 60 frames/s.

**Figure 2:**
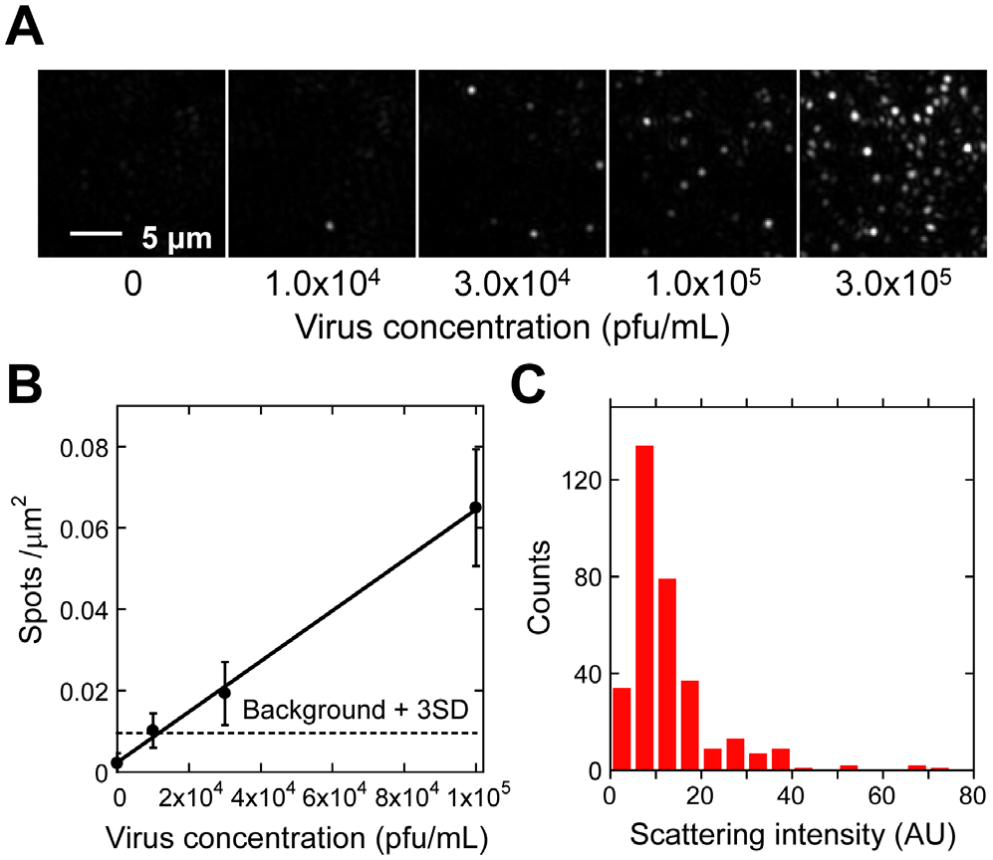
Dependence of spot density on the virus concentration as observed by TIRDFM. (A) Scattering image of viral particles at different concentrations. The virus concentrations were 0, 1.0×10^4^, 3.0×10^4^, 1.0×10^5^, and 3.0×10^5^ pfu/mL. (B) Spot density plotted against virus concentration. The LOD was found to be 1.2×10^4^ pfu/mL. (C) Distribution of the intensity of scattering spots.

### Observation of Viral Particles with TIRDFM and TIRFM

The sample chamber was constructed with 2 glass coverslips (thickness, 0.12–0.17 mm; Matsunami Glass, Tokyo, Japan), cleaned using 10 M KOH, and separated by 2 spacers that were approximately 50 µm thick. Poly-l-lysine (5 mg/mL, MW>300,000, Sigma) was infused into the sample chamber such that the surface of the coverslips was covered with it. After 20 min, the excess poly-l-lysine was washed away with PBS without Mg^2+^and Ca^2+^(PBS(-)). Then, purified viral particles diluted with PBS(-) were infused into the sample chamber and incubated at room temperature so that the viruses gradually and nonspecifically bound to the glass surface. After 1 h, the chamber was washed with PBS(-). Light scattered by the viruses on the bottom coverslip was then observed with TIRDFM.

**Figure 3:**
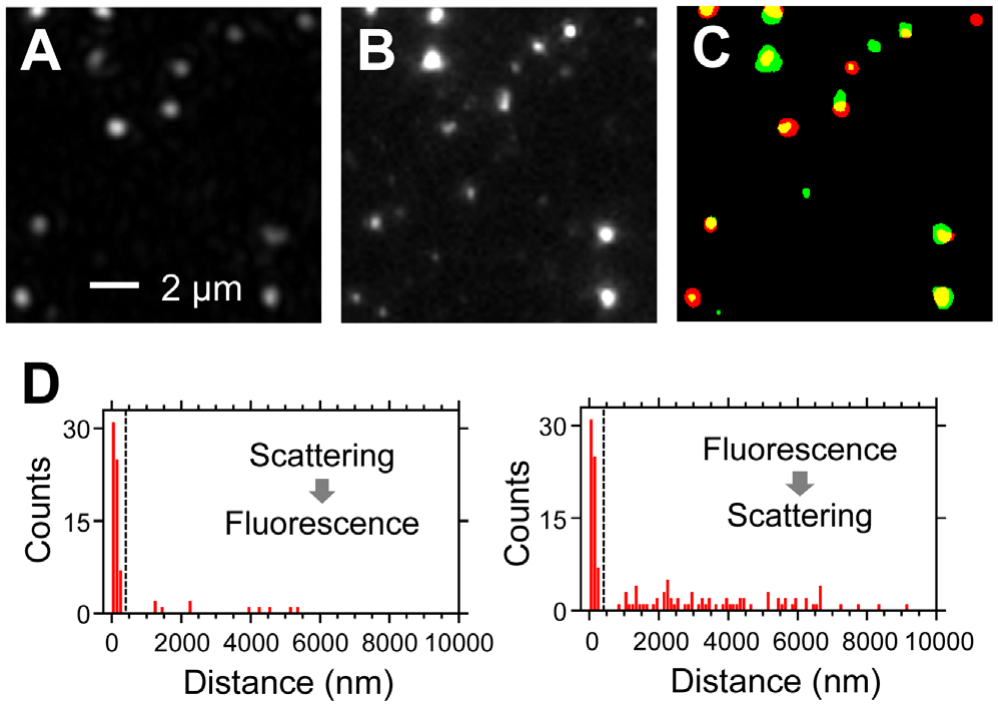
Correlation between scattering and fluorescence images of influenza viruses obtained with TIRDFM and TIRFM. (A) Scattering image. (B) Fluorescence image. (C) False-color superimposed image of binarized scattering (red) and fluorescence (green) images shown in A and B. (D) Distribution of the distance between scattering (fluorescence) spots and nearest-neighbor fluorescence (scattering) spots. We considered scattering and fluorescence spots within 400 nm (dashed line) as signals from the same particle.

For observation of the same field of view with TIRDFM and TIRFM, 1.0×10^6^ pfu/mL viral particles were mixed with 400 nM Cy3-labeled antibody at a volume ratio of 1∶1 for 4 h at room temperature; the mixture was then stored on ice for 30 h. Subsequently, it was infused into the sample chamber. After 30 min, the sample chamber was washed using PBS(-). First, Cy3-labeled antibody was observed with TIRFM. After photobleaching of Cy3, the emission filter was removed and the dichroic mirror was replaced with a perforated mirror. Light scattered by the viral particles was then observed with TIRDFM. The total observed area was 2592 µm^2^. The scattering and fluorescence images were both analyzed using the ImageJ software (particle analysis; National Institutes of Health, Bethesda, MD USA). The scattering spots were defined as spots with an area composed of consecutive pixels in which each pixel had an intensity higher than the threshold intensity of 19 arbitrary units (AUs) in 8-bit (0–255 AU) images. This intensity threshold was determined to distinguish the scattering spots from the weak background scatter and to determine the XY coordinate of the spot. Similarly, fluorescence spots were defined as a spots with an area composed of consecutive pixels in which each pixel had an intensity above the threshold intensity of 25 AU in 8-bit (0–255 AU) images. This intensity threshold was used to distinguish the fluorescent spots from the background and to determine the XY coordinate of the spot.

**Figure 4:**
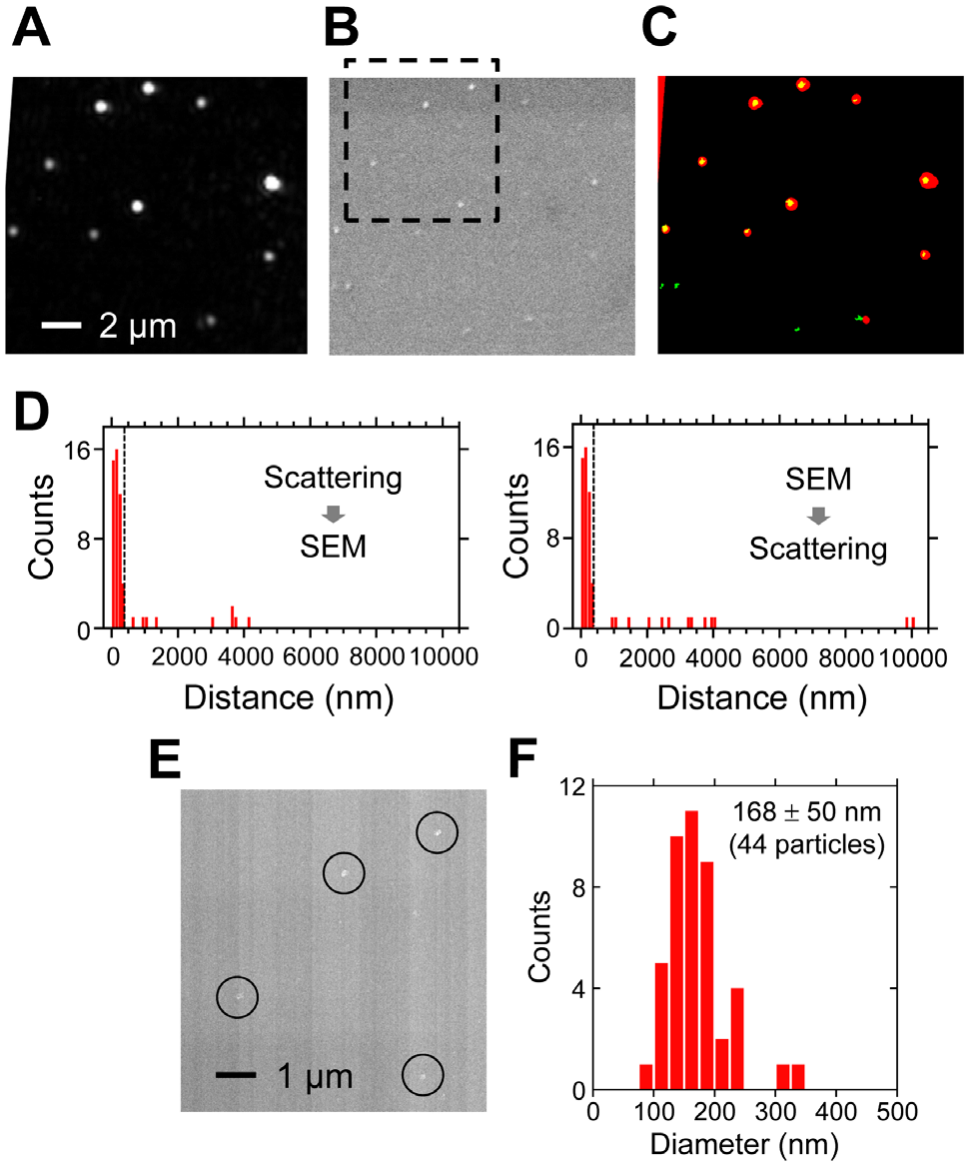
Correlation between scattering and SEM images of viruses . (A) Scattering image. (B) SEM image. (C) False-color superimposed image of binarized scattering (red) and SEM (green) images shown in A and B. (D) Distribution of the distance between scattering spots (spot in SEM) and nearest-neighbor spots in SEM (scattering spot). We considered scattering and SEM spots within 400 nm (dashed line) as signals from the same particle. (E) FE-SEM image. The area enclosed by the black dashed square in Figure 4B is shown. The particles are indicated by circles. (F) Distribution of the diameter of each particle measured in the FE-SEM images.

The dependence of density of the scattering spot observed with TIRDFM on virus concentration was measured by changing the virus concentration in sample infused into the chamber. Total observed area for each concentration was set at 6480 µm^2^. To count the scattering spots, the images were analyzed using the ImageJ software. The scattering intensity of each spot was defined as the average intensity over an 8×8-pixel area.

### Observation of Viral Particles with TIRDFM and SEM

The virus suspension (1.4×10^5^ pfu/mL) was infused into the sample chamber consisting of 2 Poly-l-lysine-coated glass coverslips. After 1 h, the excess virus suspension was washed away with PBS(-), and 2.5% glutaraldehyde was infused into the chamber so that the amino groups in the viruses were cross-linked and the viral particles were firmly immobilized on the surface of the glass coverslip. After observation with TIRDFM, the bottom coverslip was dried and coated with osmium. The sample was observed at 3,000–5,000×magnification using a scanning electron microscope with a tungsten filament as an electron source (JEOL JSM-6390SPG, JEOL Ltd, Tokyo, Japan). The locations of the areas observed with TIRDFM and SEM were identified by an etched pattern on the glass coverslip. The ImageJ sofware was used to analyze both TIRDFM and SEM images.

**Figure 5:**
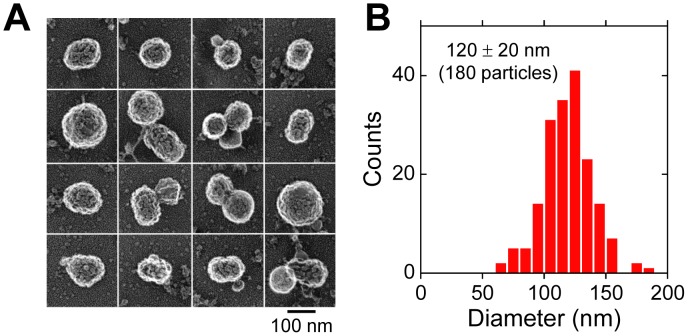
Observation of viral particles with rapid-freeze, freeze-dry, platinum-replica TEM. (A) Images of viral particles. Scale bar, 100 nm. (B) Distribution of the diameter of viral particles visualized in TEM images.

For quantitative analysis of the size of the particles, the sample observed with SEM was also observed with field-emission SEM (FE-SEM) (JSM-6335F, JEOL Ltd, Tokyo, Japan) at 8,000–15,000×magnification.

### Correlation between TIRDFM and TIRFM Images and between TIRDFM and SEM Images

The correlation between images obtained by different methods was assessed quantitatively as follows: For images obtained by TIRDFM and TIRFM, the XY coordinate of each spot in each image was calculated. Then, a single fluorescence (scattering) spot located at the nearest neighbor for a single scattering (fluorescence) spot was selected and the distance between these 2 spots was measured. To correlate the images obtained by TIRDFM and SEM, the SEM image was scaled and rotated to adjust the etched pattern markers on the glass coverslip because the magnification and orientation of images obtained by TIRDFM and SEM were different. After the adjustment, the spots in each image were analyzed as described above.

### Observation of Viruses by Rapid-freeze, Freeze-dry, Platinum Replica Transmission Electron Microscopy

The virus solution (1.4×10^6^ pfu/mL) was infused into the sample chamber, which consisted of 2 Poly-l-lysine-coated glass coverslips. After 1 h, the excess virus was washed away with PBS(-), and 2.5% glutaraldehyde in PBS(-) was infused into the chamber so that the amino groups in the viruses were cross-linked and viral particles were firmly immobilized on the surface of the glass coverslip. The sample chamber was then disassembled, and the bottom coverslip was cut into 5 mm×5 mm-pieces and washed with distilled water. The coverslip pieces were rapidly frozen by contact with a pure copper block cooled in liquid helium (Variant Instrument, USA), freeze-dried at −80°C for 15 min, and rotary-shadowed with platinum/carbon and carbon (EM-19500 JFDII, JEOL Ltd, Tokyo, Japan). The replicated virus immobilized on the surface was observed at 30,000×magnification with a conventional transmission electron microscope (TEM) (JEM1400, JEOL Ltd, Tokyo, Japan) equipped with a side-mounted CCD camera (2,048×2,048 pixels; Veleta, Olympus) [8], [9]. The total observed area was 668 µm^2^.

## Results and Discussion

### Correlation of Images of Influenza Viral Particles Obtained by TIRDFM and TIRFM

Dark-field, scattering images of the influenza virus immobilized on the glass coverslip were obtained by TIRDFM. In the absence of viral particles, only a weak background signal generated by the roughness of the glass surface or interference in the light path was obtained, and bright scattering spots were rarely observed (2.2×10^−3^ spots/µm^2^, also see Figure 2). In contrast, when the virus suspension was injected into the sample chamber, bright scattering spots appeared.

To determine whether each bright scattering spot could really be attributed to the viral particles, the locations of the scattering spots were compared with those of the fluorescence spots generated by a Cy3-labeled antibody that recognizes the influenza virus, by using the images of the same field of view obtained by TIRDFM and TIRFM. Scattering and fluorescence images were obtained for viral particles that were pre-mixed with the Cy3-labeled antibody (Figure 3A, B). A representative superimposed image of the scattering and fluorescence images is shown in Figure 3C. The locations of the most spots were almost totally consistent between the scattering and fluorescence images. Then, a single fluorescence (scattering) spot located at the nearest neighbor for a single scattering (fluorescence) spot was selected and the distance between these 2 spots was measured. The distributions of the distance had a single large peak at approximately 0–300 nm (Figure 3D). Considering the pixel size of the image (180 nm/pixel) and the lateral resolution of optical microscopy (∼220 nm), we defined scattering and fluorescence spots localized within 400 nm as signals from the same particle. Then, 86% ([63/73]×100) of the scattering spots also exhibited a fluorescence signal, which indicates that most of the scattering spots were generated by viral particles. In contrast, only 46% ([63/138]×100) of the fluorescence spots showed a scattering signal. The remaining 54% of the fluorescence spots were likely to be the result of the low scattering intensity exhibited by the fragments of the viral membrane.

### Correlation of Images of Influenza Viral Particles Obtained by TIRDFM and SEM

To further confirm that the scattering spots observed in TIRDFM represent the viral particles, the same fields of view were observed with TIRDFM and SEM. The pattern of the locations of the spots appeared to be similar for images obtained by TIRDFM and SEM (Figure 4A, B, and C). Then, a single spot observed by SEM (scattering spot) located at the nearest neighbor for a single scattering spot (spot observed by SEM) was selected and the distance between these 2 spots was measured. The distributions of the distance had a single large peak at approximately 0–400 nm (Figure 4D). We defined scattering spots and spots observed by SEM localized within 400 nm to be signals from same particle. Then, 84% ([47/56]×100) of the scattering spots corresponded to spots observed by SEM. Additionally, 78% ([47/60]×100) of the spots observed by SEM corresponded to the scattering spots. These results show good agreement between the scattering spots observed by TIRDFM and the spots observed by SEM, indicating that these spots represent viral particles.

The spots observed by conventional SEM appeared to be blurred because of low resolution; therefore, quantitative estimation of the size was not possible. The same field of view was also subsequently observed by FE-SEM, which has a higher lateral resolution than conventional SEM (Figure 4E). Although correlation analysis with the scattering spots observed by TIRDFM was difficult, because of the low contrast of the spots observed by FE-SEM and the heterogeneous background level, high-resolution images of spots with a clear edge were obtained. The spots had a size distribution of 90–350 nm (Figure 4F), and the average size was 168±50 nm (Mean ± SD, 44 particles). This value was comparable with the size of a single particle of the influenza virus [10], again supported the conclusion that these spots represent the viral particles. Furthermore, this result strongly indicated that scattering spots represent the single viral particles.

### Dispersion of Viral Particles Assessed by TEM

To further examine whether each scattering particle observed by TIRDFM corresponds to a single viral particle or particle aggregates, viral particles immobilized on a glass coverslip were observed by rapid-freeze, freeze-dry, platinum replica TEM. Many spherical particles were observed to have a rugged surface that was presumably generated by spike proteins (HA and NA) on the virus surface (Figure 5A). The average diameter of a single particle was 120±20 nm (Mean ± SD, 180 particles) (Figure 5B). This value was almost identical to the previously reported size of a single viral particle [10]. Most of the viruses existed as a single particle, whereas some formed aggregates. The fraction of singlet, doublet, triplet and larger aggregates of viral particles were 73.7%, 17.1%, 6.9%, and 2.3% of 217 particles in total, respectively.

Consistent with the results of TEM observation, the distribution of the scattering intensity of each spot obtained with TIRDFM showed a single peak with a small population with a higher intensity (Figure 2C). The scattering intensity depends strongly on the radius *r* of the scattering object and is proportional to *r* ^6^ [11]. The lower variation in the scattering intensity further validates that most scattering spots were derived from single viral particles.

### Limit of Detection of TIRDFM for Viral Particles

The density of the scattering spots at different virus concentrations was analyzed (Figure 2A). The spot density proportionally decreased with decrease in the virus concentration. The linear fitting is shown in Figure 2B. The LOD for spot detection was determined from the extrapolation of the virus concentration at a spot density equal to the background density (2.2×10^−3^ spots/µm^2^) plus 3 times the standard deviation (SD) of the background (Figure 2B). The LOD reached 1.2×10^4^ pfu/mL, which is comparable to the LOD of the influenza virus determined using ELISA [12].

In our experiment, concentration of viruses with infectious ability was determined in advance by plaque formation assay. When virus concentration was 1.0×10^5^ pfu/mL, the spot density of bound viruses was 0.065 spots/µm^2^ (Figure 2B). Considering the volume of the sample chamber (5 µL) and the surface area of the coverglass, 1.0×10^5^ pfu/mL would correspond to the density of only 3.1×10^−5^ viruses/µm^2^, if we assume that all virus particles have infectious ability and are bound to the glass surface. Therefore, the density of scattering spots was approximately ∼2000 times higher than that of viruses with infectious ability. It is known that most of influenza virus particles cannot accomplish infection because of defective infectious process including 8-genome packing. To understand the mechanism of infection and efficiency of infection, it is important to investigate the ratio of particles that have infectious ability by counting the influenza virus particle. In the previous studies using electron microscopy, the ratio of the observed particles to the particles with infectious ability was reported to be 10−100 [13]. These values are much smaller than that estimated by our method (∼2000). The differences may be caused by the degree of defective viruses during formation, the degree of damage during purification and storage, and the different conditions of the plaque formation assay.

### Conclusion

In this study, we performed label-free imaging of single particles of the influenza virus with TIRDFM. The optical system for TIRDFM is simpler than other systems reported previously [1]–[4]. The LOD for the viral particle reached 1.2×10^4^ pfu/mL, which is comparable to that for ELISA. The LOD can be further improved by selective immobilization of the virus on the glass coverslip by using an antibody. Specific binding of the virus to the glass surface may also permit virus detection in saliva and nasal mucus with TIRDFM. In addition, other viruses, typically those that are larger than the influenza virus, may also be detected with TIRDFM.
